# Trust and Psychosomatic Complaints in Adolescence: Findings From a Swedish Cohort Study

**DOI:** 10.3389/ijph.2023.1606032

**Published:** 2023-10-11

**Authors:** Sara Brolin Låftman, Viveca Östberg, Jonas Raninen

**Affiliations:** ^1^ Department of Public Health Sciences, Centre for Health Equity Studies (CHESS), Stockholm University, Stockholm, Sweden; ^2^ Department of Clinical Neuroscience, Karolinska Institutet, Stockholm, Sweden; ^3^ Centre for Alcohol Policy Research, La Trobe University, Melbourne, VIC, Australia

**Keywords:** generalised trust, institutional trust, psychosomatic complaints, health complaints, family characteristics

## Abstract

**Objectives:** To examine the cross-sectional and longitudinal associations between generalised and institutional trust and psychosomatic complaints in mid and late adolescence.

**Methods:** Data were derived from the Swedish cohort study Futura01, using survey information collected amongst 3,691 grade 9 students (∼15–16 years, t1) who were followed-up 2 years later (∼17–18 years, t2). Registry information on sociodemographic characteristics was linked to the data. Linear regression analyses were performed. The longitudinal analyses applied the first difference (FD) approach as well as the lagged dependent variable (LDV) approach. Covariates included gender, family type, parental education, parental country of birth, and upper secondary programme.

**Results:** Higher levels of generalised and institutional trust were cross-sectionally associated with lower levels of psychosomatic complaints at both time points. The FD analyses showed that increases in generalised and in institutional trust between ages 15–16 and 17–18 years were associated with corresponding decreases in psychosomatic complaints. The LDV analyses demonstrated reciprocal temporal associations between trust and psychosomatic complaints.

**Conclusion:** The findings indicate that trust is a social determinant of psychosomatic complaints in adolescents, but also that health may affect trust.

## Introduction

Trust is a key component of a well-functioning and socially sustainable society [1–3], not least due to its links with good health and wellbeing, which are also essential aspects of sustainable development [4]. A conceptual distinction can be made between trust in other people (commonly referred to as generalised; horisontal; social or interpersonal trust), and trust in public institutions (commonly referred to as institutional or vertical trust) [5]. At the individual level, these two dimensions of trust are empirically related with one another [6, 7].

A plethora of research has demonstrated associations between trust in other people and various health outcomes among adults, with lower levels of trust being related to a higher risk of morbidity [8–13] and mortality [14, 15]. One proposed mechanism is that higher levels of trust enhance social support and collective action, which may aid people to better cope with stressors [10, 15]. Low trust in other people can also be regarded as a stressor in itself, affecting individuals’ health through psychosocial pathways [15]. Fewer studies have focused specifically on the association between trust in public institutions and health [16]. It has been shown that political trust (which is one aspect of trust in public institutions), is positively associated with self-rated health [17] and psychological health [18], even when adjusting for trust in other people. Low trust in public institutions may, just as low trust in other people, be a stressor with implications for health [18]. However, it has also been postulated that the association may be due to reversed causality, in that poorer health could lead to lower political trust [19].

Trust tends to be developed and shaped early in life [20, 21], partly though parental socialisation [22] but also via other social contexts such as the school [23, 24]. Although there is evidence of instability in trust in mid-adolescence, it seems to stabilise with age [25]. Some studies have examined the associations between trust and self-reported health in young people. Tuominen and Haanpää [26] demonstrated that cross-sectionally there was a link between trust in other people and higher life satisfaction in a Finnish sample of 12–13 years-olds. In a longitudinal study focusing on young adults in Stockholm, Winzer et al. [27] demonstrated that trust in other people was a determinant of stable mental health, whereas trust in the community was not. In their cross-sectional study of disadvantaged Baltimore youth, Mmari et al. [28] did not report any statistically significant differences in self-rated health by community trust or institutional trust. Additionally, a body of research has examined the links between concepts associated with trust and young people’s wellbeing and health. For instance, higher levels of wellbeing and better health have been reported among adolescents with higher levels of social capital [29], general belonging [30], sense of unity [31], sense of community [32, 33], and connectedness [34, 35]. Taken together, however, longitudinal studies into the links between different dimensions of trust and health in adolescents are sparse.

Adolescence and the transition into adulthood are life phases characterised by significant development and changes [36, 37]. This period is sometimes referred to as “emerging adulthood,” which covers the mid-teen years to the mid or late twenties [38]. Emerging adulthood is regarded as a critical period due to the high degree of instability and the many challenges faced [37, 39]. While physical health is generally good in this period of life, psychosomatic complaints are common, especially among girls [40]. The prevalence of psychosomatic complaints has also been shown to vary by other sociodemographic characteristics than gender, including family type, family affluence, and foreign background [41]. Psychosomatic complaints are correlated with perceived stress and can hence be regarded as stress-related [41].

The aim of the current study was to examine the cross-sectional and longitudinal associations between generalised trust (i.e., trust in other people) and institutional trust (i.e., trust in public institutions) and psychosomatic complaints in mid and late adolescence.

## Methods

### Data Material

Data were derived from the Swedish cohort study Futura01, which was based on a national sample of adolescents attending grade 9, i.e., the final grade of compulsory school, in spring 2017 (∼15–16 years). A random sample of 500 schools across Sweden was drawn, and one class in each school was selected. In total, 343 schools agreed to participate, rendering a school level response rate of 69% [42]. There were no statistically significant differences between participating and non-participating schools with regards to average grades, the proportion of students with highly educated parents, or the proportion of students with foreign-born parents [42]. The first data collection (t1) was carried out in classrooms in 2017 with a paper-and-pencil questionnaire (*n* = 5,537; response rate 82%). The second wave (t2) was performed in 2019 (when respondents typically attended the second grade of upper secondary school; ∼17–18 years) as a web and postal survey (*n* = 4,141; i.e., 75% of those participating at t1). More information on the data collection and material is provided elsewhere [42]. Official register information on parental education and parental country of birth has been matched to the survey data. The linkage was performed by Statistics Sweden. The data material used for the current study was deidentified. The analytical sample includes individuals with information on all study variables at t1 and t2 (*n* = 3,691; i.e., 67% of those participating at t1). Ethical approval has been obtained from the Swedish Ethical Review Authority (ref. 2021-06504-01; 2022-02781-02). The participants provided informed written consent.

### Measures

Psychosomatic complaints were measured (at t1 and t2) by the question: “During the past 6 months, how often have you had…” and the items a) “headache,” b) “stomach ache,” and c) “difficulties falling asleep.” The response categories were “Every day,” “A few times a week,” “Once a week,” “Some time a month,” and “Less often or never.” The internal consistency of the items was acceptable given the small number of items (Cronbach’s alpha t1: 0.64; t2: 0.63). The variable was based on information from participants who had responded to all three items. The values of the three items were added to a summary measure with the range 3–15, with higher values representing more frequent complaints. The same set of items have been used previously to measure psychosomatic complaints [43–46].

The items on generalised and institutional trust were retrieved from the OECD measurement of social capital project and question databank [47] and subsequently adjusted to the target population. They have been used in a prior study based on the Futura01 data [48].

Generalised trust was measured (at t1 and t2) by the opening question: “Considering society as a whole, mark the alternative that best agrees with how you feel,” and the items a) “You can trust most people”; b) “You can never be too careful when you meet new people”; c) “Most people are trying to be helpful”; d) “Most people only care about themselves”; and e) “Most people are honest.” The response categories were “Totally correct”; “Partly correct”; “Partly incorrect”; and “Totally incorrect,” and were assigned the values 4, 3, 2, and 1 (items b and d were reversely coded). The five items showed acceptable internal consistency (Cronbach’s alpha t1: 0.62; t2: 0.68). In accordance with a previous study based on the same data [48], removing item b improved the internal consistency (Cronbach’s alpha t1: 0.72; t2: 0.74). Hence, we calculated the mean value of items a, c, d, and e for participants who responded to at least three of these. The range of the measure was 1–4, with higher values indicating higher levels of generalised trust.

Institutional trust was measured (at t1 and t2) by the opening question: “How much do you normally trust…,” and the items a) “Government and parliament”; b) “The justice system (police and courts)”; c) “Teachers”; d) “News (TV, radio)”; and e) “Researchers and experts.” The response categories were “Very much”; “Fairly much”; “Not that much”; and “Not at all,” and were assigned the values 4, 3, 2, and 1. The items showed high internal consistency (Cronbach’s alpha t1 = 0.75; t2 = 0.73). For respondents who answered at least three of the five items, the mean value of the items was calculated to indicate institutional trust. The range of the measure was 1–4, with higher values indicating higher levels of institutional trust. The correlation between generalised and institutional trust was moderate (Pearson’s r t1: 0.35; t2: 0.45).

A set of control variables were included to adjust for sociodemographic characteristics.

Gender was based on information from the participants’ personal security numbers used to indicate the categories boys and girls.

Family type was measured (at t1) by the question: “How do you live?” and the response categories “Lives with mother and father”; “Lives with mother”; “Lives with father”; and “Lives about half of the time with mother and about half of the time with father (shared residence).” A variable was constructed distinguishing between participants living with two parents, with single parent, in shared residence, and others/missing.

Parental education was derived from official register information on father’s and mother’s educational level from 2017. Three categories were formed, based on the highest educational level among parents: upper secondary school ≤2 years or less; upper secondary school ≥3 years; and tertiary education.

Parental country of birth was based on official register information on father’s and mother’s country of birth. A variable was constructed to distinguish between at least one parent born in Sweden; at least one parent born in Europe; and two parents born outside Europe.

Upper secondary programme was measured (at t2) by the question: “What orientation does your in upper secondary school programme have?” The response categories were “Vocational,” “Academic,” and “Other.” Participants who attended an “other” programme, those who did not go to upper secondary school, and those with missing information were coded into one category.

### Statistical Analysis

To examine the associations that generalised and institutional trust share with psychosomatic complaints, we carried out both cross-sectional and longitudinal linear (OLS) regression analyses. The independent variables of interest—generalised and institutional trust—were continuous, whereas the covariates were categorical.

The cross-sectional associations between generalised and institutional trust and psychosomatic complaints at t1 and at t2, respectively, were analysed through a series of linear regression models, presenting unstandardised b coefficients and 95% confidence intervals. To evaluate the longitudinal associations between generalised and institutional trust and psychosomatic complaints, linear regressions were performed using the first difference (FD) approach [49], analysing the change in generalised and in institutional trust (t2−t1) predicting change in psychosomatic complaints (t2−t1). A strength of the FD method is that it takes time-invariant confounding into account [49]. In both the cross-sectional and the longitudinal FD analyses of trust and psychosomatic complaints, we first performed a crude model, including only one independent/control variable at a time whilst adjusting for gender. Model(s) 1 included generalised trust and all control variables. Model(s) 2 included institutional trust and all control variables. Model(s) 3 included both generalised and institutional trust as well as all control variables. We also tested for interactions between generalised and institutional trust and the covariates. Wald tests were used to compare the model fit with and without the interaction terms. All statistically significant interactions (*p* < 0.05) are reported in the tables.

Finally, to gain further insight into the temporal direction between generalised and institutional trust and psychosomatic complaints, we performed a set of linear regression models using the lagged dependent variable (LDV) approach, regressing the outcome on the predictors whilst controlling for the baseline value of the outcome [49]. As a first step of the LDV analysis, psychosomatic complaints at t2 was treated as the dependent variable in linear regression models, and generalised and institutional trust at t1 were treated as predictors, controlling also for psychosomatic complaints at t1. As a second step of the LDV analysis, generalised and institutional trust at t2 were treated as dependent variables in linear regression models and psychosomatic complaints at t1 as the predictor, controlling also for generalised/institutional trust at t1. In the LDV analyses, we used z-standardised measures of trust and psychosomatic complaints to facilitate comparison of estimates.

To account for the hierarchical nature of the data with students nested in school classes at t1, robust standard errors were estimated clustering by class at t1, using the “cluster” option in Stata. The number of classes was 335. All statistical analyses were performed with Stata, version 17 [50].

## Results

Descriptives of the analytical sample are presented in Table 1. The analytical sample comprised of 44.7% boys and 55.3% girls. While 70.4% lived with two parents in one household, 13.7% lived with a single parent, 13.6% in shared residence and 2.3% in other family constellations (or had missing information on the variable). With regards to parental education, 15.1% had parents with ≤2 years upper secondary education or less, 20.4% had at least one parent with ≥3 upper secondary education, and 64.5% had at least one parent with tertiary education. A majority (85.0%) had at least one parent born in Sweden, 5.0% had at least one parent born in Europe, and 10.0% had two parents born outside Europe. Among the participants, 20.9% attended a vocational programme and 75.8% attended an academic programme at t2. A small proportion (3.3%) attended another upper secondary programme, did not go to upper secondary school, or had missing information on the variable. The mean value for psychosomatic complaints was 7.05 at t1 and 7.25 at t2. The mean change in psychosomatic complaints (t2−t1) was 0.20. The mean value for generalised trust was 2.41 at t1 and 2.42 at t2, and the mean change (t2−t1) was 0.01. The mean value for institutional trust was 2.82 at t1 and 2.80 at t2, with a mean change (t2−t1) of −0.02.

**TABLE 1:** Descriptives *n* = 3,691. Futura01 survey, Sweden, 2017 and 2019.

	*n*	%				
Gender						
Boys	1,651	44.7				
Girls	2,040	55.3				
Family type (t1)						
Two parents	2,598	70.4				
One parent	506	13.7				
Shared residence	503	13.6				
Other/missing	84	2.3				
Parental education						
≤2 years secondary or less	557	15.1				
≥3 years secondary	754	20.4				
Tertiary	2,380	64.5				
Parental country of birth						
At least one in Sweden	3,139	85.0				
At least one in Europe	183	5.0				
Two parents outside Europe	369	10.0				
Upper secondary programme (t2)						
Vocational	772	20.9				
Academic	2,796	75.8				
Other programme/other activity/missing	123	3.3				
	Mean	s.d.	Min.	Max.	Skewness	Kurtosis
Psychosomatic complaints						
t1	7.05	2.75	3	15	0.52	2.55
t2	7.25	2.72	3	15	0.43	2.49
Change (t2−t1)	0.20	2.45	−10	12	0.04	3.86
Generalised trust						
t1	2.41	0.51	1	4	−0.18	2.97
t2	2.42	0.51	1	4	−0.19	2.94
Change (t2−t1)	0.01	0.52	−2.25	1.75	−0.07	3.69
Institutional trust						
t1	2.82	0.56	1	4	−0.50	3.54
t2	2.80	0.54	1	4	−0.45	3.48
Change (t2−t1)	−0.02	0.55	−3.00	2.80	−0.03	4.58

Descriptives of the full t1 sample are presented in the Supplementary Table S1. Comparing the analytical sample with the full t1 sample indicated some systematic bias in individual drop-out between the two data collections (and/or in non-complete answers): in the analytical sample, there was a slight underrepresentation of boys, of adolescents not living with two parents, of adolescents whose parents had a lower education, of those with two parents born abroad, and of those not attending an academic upper secondary programme at t2. Mean values of generalised and institutional trust by the covariates and results from ANOVAs are presented in the Supplementary Table S2. The results show clear differences in levels of trust between groups of adolescents.

Results from the cross-sectional analyses of psychosomatic complaints at t1 regressed on generalised and institutional trust at t1 are presented in Table 2, and results from t2 are presented in Table 3. The analyses of data from t1 (Table 2) showed that higher levels of trust were associated with lower levels of psychosomatic complaints. This was true for the crude analyses and Models 1–2, including one form of trust at a time, as well as in the fully adjusted analysis of Model 3 although the estimates were somewhat attenuated (generalised trust: *b* = −0.83, 95% CI −1.00, −0.67; institutional trust: *b* = −0.85, 95% CI −1.00, −0.71). With regards to the covariates, the fully adjusted model (Model 3) showed that girls reported higher levels of psychosomatic complaints than boys. The same was true for adolescents who lived with a single parent and those with shared residence compared with those who lived in two parent households. There were no statistically significant differences in psychosomatic complaints by parental education in Model 3. Adolescents with parents born in Europe or outside Europe reported lower levels of psychosomatic complaints compared with adolescents with those with at least one parent born in Sweden. Interaction analyses (not presented in Table) detected two statistically significant interactions, indicating that trust was more strongly associated with psychosomatic complaints among girls than among boys (generalised trust*gender: *p* = 0.004; institutional trust*gender: *p* < 0.001). The results from t2 echo those from t1 with the patterning in Table 3 almost identical to that in Table 2, with the exception for parental country of birth not being significant. Upper secondary programme at t2 was included in the analyses of t2 only. Adolescents attending an academic programme reported fewer psychosomatic complaints than those attending a vocational programme in the crude analysis, but not in the adjusted models (Models 1–3).

**TABLE 2:** Results from cross-sectional linear regression analyses of psychosomatic complaints at t1 by generalised and institutional trust at t1 (age 15–16 years). *n* = 3,691. Futura01 survey, Sweden, 2017.

	Psychosomatic complaints (t1)							
	Crude^a^		Model 1^b^		Model 2^c^		Model 3^d^	
	b	95% CI	b	95% CI	b	95% CI	b	95% CI
Generalised trust (t1)	−1.21***	−1.37, −1.05	−1.15***	−1.31, −0.99			−0.83***	−1.00, −0.67
Institutional trust (t1)	−1.18***	−1.32, −1.04			−1.11***	−1.25, −0.97	−0.85***	−1.00, −0.71
Gender								
Boys (ref.)	0.00	—	0.00	—	0.00	—	0.00	—
Girls	1.69***	1.51, 1.87	1.56***	1.39, 1.73	1.71***	1.54, 1.87	1.63***	1.46, 1.79
Family type (t1)								
Two parents	0.00	—	0.00	—	0.00	—	0.00	—
One parent	1.12***	0.87, 1.36	0.89***	0.64, 1.14	0.93***	0.68, 1.17	0.84***	0.59, 1.08
Shared residence	0.53***	0.28, 0.78	0.42**	0.16, 0.67	0.44**	0.19, 0.69	0.40**	0.14, 0.65
Other/missing	0.87**	0.24, 1.51	0.65*	0.03, 1.28	0.62	0.00, 1.25	0.55	−0.07, 1.18
Parental education								
≤2 years secondary or less	0.06	−0.22, 0.34	0.07	−0.21, 0.34	0.01	−0.26, 0.28	0.03	−0.24, 0.29
≥3 years secondary (ref.)	0.00	—	0.00	—	0.00	—	0.00	—
Tertiary	−0.42***	−0.63, −0.20	−0.25*	−0.46, −0.05	−0.14	−0.34, 0.06	−0.13	−0.33, 0.07
Parental country of birth								
At least one in Sweden (ref.)	0.00	—	0.00	—	0.00	—	0.00	—
At least one in Europe	−0.22	−0.59, 0.16	−0.51*	−0.90, −0.12	−0.43*	−0.81, −0.05	−0.55**	−0.93, −0.17
Two parents outside Europe	−0.15	−0.47, 0.18	−0.39*	−0.71, −0.07	−0.30	−0.62, 0.01	−0.40*	−0.71, −0.09
Generalised trust*gender								*p* = 0.004
Institutional trust*gender								*p* < 0.001

***p < 0.001, **p < 0.01, *p < 0.05.

^a^ Includes one independent variable at a time, controlling for gender.

^b^ Includes generalised trust and all covariates.

^c^ Includes institutional trust and all covariates.

^d^ Includes generalised and institutional trust and all covariates.

**TABLE 3:** Results from cross-sectional linear regression analyses of psychosomatic complaints at t2 by generalised and institutional trust at t2 (age 17–18 years). *n* = 3,691. Futura01 survey, Sweden, 2019.

	Psychosomatic complaints (t2)							
	Crude^a^		Model 1^b^		Model 2^c^		Model 3^d^	
	b	95% CI	b	95% CI	b	95% CI	b	95% CI
Generalised trust (t2)	−1.14***	−1.31, −0.97	−1.04***	−1.22, −0.86			−0.66***	−0.85, −0.46
Institutional trust (t2)	−1.24***	−1.40, −1.09			−1.15***	−1.32, −0.99	−0.89***	−1.06, −0.71
Gender								
Boys (ref.)	0.00	—	0.00	—	0.00	—	0.00	—
Girls	1.62***	1.45, 1.79	1.56***	1.40, 1.72	1.66***	1.50, 1.82	1.62***	1.46, 1.78
Family type (t1)								
Two parents	0.00	—	0.00	—	0.00	—	0.00	—
One parent	1.03***	0.79, 1.27	0.75***	0.50, 0.99	0.78***	0.54, 1.02	0.70***	0.46, 0.94
Shared residence	0.52***	0.30, 0.75	0.40**	0.17, 0.63	0.43***	0.21, 0.66	0.38**	0.15, 0.60
Other/Missing	0.58*	0.02, 1.14	0.26	−0.30, 0.83	0.35	−0.23, 0.92	0.25	−0.32, 0.81
Parental education								
≤2 years secondary or less	0.09	−0.22, 0.39	0.00	−0.31, 0.30	−0.03	−0.33, 0.27	−0.02	−0.32, 0.28
≥3 years secondary (ref.)	0.00	—	0.00	—	0.00	—	0.00	—
Tertiary	−0.41***	−0.63, −0.19	−0.17	−0.39, 0.04	−0.12	−0.33, 0.09	−0.09	−0.31, 0.12
Parental country of birth								
At least one in Sweden (ref.)	0.00	—	0.00	—	0.00	—	0.00	—
At least one in Europe	0.20	−0.18, 0.58	−0.08	−0.46, 0.31	−0.04	−0.42, 0.33	−0.14	−0.51, 0.24
Two parents outside Europe	0.20	−0.10, 0.50	−0.13	−0.43, 0.18	−0.03	−0.34, 0.28	−0.17	−0.48, 0.14
Upper secondary programme (t2)								
Vocational	0.00	—	0.00	—	0.00	—	0.00	—
Academic	−0.39***	−0.60, −0.17	−0.16	−0.38, 0.06	0.01	−0.20, 0.23	0.00	−0.21, 0.22
Other programme/other activity/missing	0.52	−0.01, 1.05	0.45	−0.05, 0.95	0.36	−0.14, 0.86	0.38	−0.11, 0.87
Generalised trust*gender								*p* = 0.003
Institutional trust*gender								*p* = 0.001

***p < 0.001 **p < 0.01 *p < 0.05.

^a^ Includes one independent variable at a time, controlling for gender.

^b^ Includes generalised trust and all covariates.

^c^ Includes institutional trust and all covariates.

^d^ Includes generalised and institutional trust and all covariates.

Results from the FD analyses, examining the change in psychosomatic complaints (t2−t1) regressed on the changes in generalised trust (t2−t1) and in institutional trust (t2−t1), respectively, are presented in Table 4. The crude analyses and Models 1–2, including one dimension of trust at a time, showed that increases in trust were associated with decreases in psychosomatic complaints. Also the analyses simultaneously adjusting for change in both dimensions of trust (Model 3) presented statistically significant, inverse associations between the change in generalised trust and the change in psychosomatic complaints (*b* = −0.29, 95% CI −0.45, −0.12), and between the change in institutional trust and the change in psychosomatic complaints (*b* = −0.44, 95% CI −0.61, −0.27). The estimates of the covariates in Model 3 indicated that the change in psychosomatic complaints between t1 and t2 did not differ by gender, family type, or parental education. However, the estimates for parental country of birth indicated that psychosomatic complaints increased more between t1 and t2 in adolescents with one parent born outside Europe, compared with those with at least one Swedish born parent. Additionally, there was a statistically significant difference in change between those attending a vocational and an academic upper secondary programme. Further analyses (not presented in Table) indicate an increase in psychosomatic complaints between t1 and t2 only in the latter category. There were no statistically significant interactions between changes in trust and the covariates.

**TABLE 4:** Results from linear regression analyses of change in psychosomatic complaints (t2−t1) by change in generalised trust (t2−t1) and change in institutional trust (t2−t1) (between age 15–16 and age 17–18 years). *n* = 3,691. Futura01 survey, Sweden, 2017 and 2019.

	Change in psychosomatic complaints (t2−t1)							
	Crude^a^		Model 1^b^		Model 2^c^		Model 3^d^	
	*b*	95% CI	*b*	95% CI	*b*	95% CI	*b*	95% CI
Change in generalised trust (t2−t1)	−0.39***	−0.56, −0.23	−0.39***	−0.55, −0.23			−0.29**	−0.45, −0.12
Change in institutional trust (t2−t1)	−0.51***	−0.68, −0.35			−0.50***	−0.66, −0.34	−0.44***	−0.61, −0.27
Gender								
Boys (ref.)	0.00	—	0.00	—	0.00	—	0.00	—
Girls	−0.07	−0.23, 0.10	−0.07	−0.24, 0.09	−0.08	−0.24, 0.08	−0.07	−0.23, 0.09
Family type (t1)								
Two parents	0.00	—	0.00	—	0.00	—	0.00	—
One parent	−0.09	−0.33, 0.15	−0.09	−0.33, 0.14	−0.10	−0.33, 0.14	−0.10	−0.34, 0.13
Shared residence	−0.01	−0.24, 0.23	0.02	−0.21, 0.26	0.03	−0.21, 0.26	0.02	−0.22, 0.25
Other/missing	−0.30	−0.90, 0.31	−0.29	−0.88, 0.31	−0.24	−0.85, 0.36	−0.26	−0.86, 0.34
Parental education								
≤2 years secondary or less	0.02	−0.30, 0.34	−0.03	−0.34, 0.29	−0.02	−0.33, 0.30	−0.02	−0.33, 0.29
≥3 years secondary (ref.)	0.00	—	0.00	—	0.00	—	0.00	—
Tertiary	0.01	−0.21, 0.23	−0.04	−0.27, 0.19	−0.05	−0.27, 0.18	−0.04	−0.26, 0.19
Parental country of birth								
At least one in Sweden (ref.)	0.00	—	0.00	—	0.00	—	0.00	—
At least one in Europe	0.42*	0.05, 0.78	0.41*	0.04, 0.79	0.41*	0.03, 0.78	0.40*	0.03, 0.78
Two parents outside Europe	0.35*	0.05, 0.66	0.30	−0.01, 0.61	0.31*	0.01, 0.61	0.28	−0.02, 0.58
Upper secondary programme (t2)								
Vocational	0.00	—	0.00	—	0.00	—	0.00	—
Academic	0.25*	0.06, 0.45	0.26*	0.06, 0.47	0.24*	0.04, 0.44	0.25*	0.05, 0.45
Other programme/other activity/missing	−0.14	−0.69, 0.41	−0.14	−0.69, 0.40	−0.16	−0.68, 0.37	−0.16	−0.68, 0.36

***p < 0.001, **p < 0.01, *p < 0.05.

^a^ Includes one independent variable at a time, controlling for gender.

^b^ Includes change in generalised trust and all covariates.

^c^ Includes change in institutional trust and all covariates.

^d^ Includes change in generalised and institutional trust and all covariates.

Finally, to examine the temporal directionality of the associations between trust and psychosomatic complaints, we also performed two pairs of linear regression analyses applying the LDV approach (using z-standardised measures of trust and of psychosomatic complaints). The results are presented in Table 5. In the first pair of analyses, psychosomatic complaints at t2 was treated as the dependent variable. The results show that higher levels of generalised trust at t1 were associated with lower levels of psychosomatic complaints at t2 (*b* = −0.05, 95% CI −0.08, −0.02). Similarly, higher levels of institutional trust at t1 were associated with lower levels of psychosomatic complaints at t2 (*b* = −0.04, 95% CI −0.06, −0.01). Furthermore, psychosomatic complaints at t1 significantly predicted psychosomatic complaints at t2. In the next pair of analyses, generalised and institutional trust at t2 were used as the dependent variables, and regressed on psychosomatic complaints at t1, controlling also for generalised/institutional trust at t1. These analyses showed statistically significant associations between psychosomatic complaints at t1 and both generalised trust (*b* = −0.07, 95% CI −0.11, −0.04) and institutional trust (*b* = −0.09, 95% CI −0.12, −0.06) at t2.

**TABLE 5:** Results from linear regression analyses of the associations between trust (standardised) and psychosomatic complaints (standardised) measured at different time points. Futura01 survey, Sweden, 2017 and 2019.

	Psychosomatic complaints (t2)	
	*b*	95% CI
Generalised trust (t1)	−0.05***	−0.08, −0.02
Psychosomatic complaints (t1)	0.54***	0.51, 0.57

	Psychosomatic complaints (t2)	
	*b*	95% CI
Institutional trust (t1)	−0.04*	−0.06, −0.01
Psychosomatic complaints (t1)	0.55***	0.52, 0.57

	Generalised trust (t2)	
	*b*	95% CI
Psychosomatic complaints (t1)	−0.07***	−0.11, −0.04
Generalised trust (t1)	0.44***	0.41, 0.47

	Institutionalised trust (t2)	
	*b*	95% CI
Psychosomatic complaints (t1)	−0.09***	−0.12, −0.06
Institutionalised trust (t1)	0.43***	0.39, 0.46

***p < 0.001, **p < 0.01, *p < 0.05.

All analyses adjusted for gender, family type, parental education, parental country of birth, and upper secondary programme. *n* = 3,691.

## Discussion

This study examined the cross-sectional and longitudinal associations between generalised and institutional trust and psychosomatic complaints in a Swedish national sample of adolescents. The data were collected in the ninth and final grade of compulsory school (age 15–16) and when adolescents typically attended the second grade of upper secondary school (age 17–18).

All of our analyses point in the same direction and paint a consistent picture of an association between trust and psychosomatic complaints. The cross-sectional analyses showed that both generalised and institutional trust was inversely associated with psychosomatic complaints at age 15–16 and at age 17–18 years, i.e., those with higher levels of trust have less psychosomatic complaints. The longitudinal analyses applying the first difference (FD) approach [49] showed that increases in trust between ages 15–16 and 17–18 years were associated with corresponding decreases in psychosomatic complaints. Even though the levels of generalised and institutional trust differed between groups of adolescents, the general absence of statistically significant interaction terms indicate that the associations between trust and psychosomatic complaints were valid for the sample as a whole. One exception was that the cross-sectional associations between trust and psychosomatic complaints were stronger for girls than for boys.

In both the cross-sectional and the longitudinal analyses, both dimensions of trust showed independent associations with psychosomatic complaints. The findings reflect prior research on trust and health amongst young people [26] and adults [8–15]. Notwithstanding, earlier research has postulated that the association between trust and health may also operate in the opposite direction, namely, that health predicts trust [19]. Therefore, as the FD approach does not inform about the temporal order of the associations, we also performed a set of analyses using the lagged dependent variable (LDV) approach [49]. These analyses showed that the levels of trust at t1 were predictive of psychosomatic complaints at t2, even with control for the baseline levels of psychosomatic complaints. However, our reversed analyses showed that psychosomatic complaints at t1 were also predictive of trust at t2, suggesting that the associations between trust and psychosomatic complaints operate in both directions.

Taken together, our findings indicate that the processes linking trust with psychosomatic complaints are reciprocal, and therefore interpretations of the associations in both directions are relevant. The possible mechanisms in the associations between generalised trust and psychosomatic complaints may relate to the assumptions that generalised trust promotes social support, which can be beneficial for health [10], and that low trust can be a stressor [15]. Such an interpretation aligns well with the findings of a mixed methods study of generalised trust among upper secondary students in Stockholm [51]. In the qualitative part of that study, the participants were asked to write letters about generalised trust. The explorative content analysis showed that the students described generalised trust as something that enhances wellbeing, and is important for making friends, for feeling safe, for not missing out on opportunities, and for more thriving on a personal level. Conversely, distrust of other people was described as tiring and stressful and associated with poorer relations with others [51]. With regards to the associations between psychosomatic complaints and later trust, one mechanism may be that stress-related health is linked with frustration, which in turn can imply more negative evaluations of people in general and of the public institutions of society [19]. It is also likely that health problems can limit individuals’ possibility to engage in social relations and in society at large [19], which may in turn impact their levels of trust.

The present study’s finding that institutional trust was inversely associated with psychosomatic complaints even when adjusting for generalised trust reflects prior research on adults [17, 18]. One interpretation is that not only low generalised trust, but also low institutional trust, can be a stressor [18]. Furthermore, low political trust has been shown to correlate with external locus of control in terms of a lack of belief in the possibility to influence one’s own health [16]. External sense of control is, in turn, indicative of, e.g., depression [52]. It is however not evident that external locus of control is a mediator on the hypothesised causal pathway between institutional trust and psychosomatic complaints, but it could possibly also be a confounder. A relevant task for future research is to delve deeper into the mechanisms in the associations between generalised and institutional trust and health among adolescents. Additionally, it would be relevant to examine the reciprocal links between trust and health using data from more than two time points.

While our analytical strategy included the FD method, examining the associations between changes in trust and changes in psychosomatic complaints, a related result that deserves to be highlighted is the relative stability of both trust and psychosomatic complaints between ages 15–16 and 17–18 in our data. The mean values and standard deviations indicate that, at the group level, the average changes were minor. With regards to the stability of trust, one interpretation is that both generalised and institutional trust are largely shaped already before mid-adolescence. This is in line with prior studies suggesting that trust tends to be formed early in life [20, 21]. Nonetheless, it should be acknowledged that the mean values conceal that there were also individuals with a substantial degree of change in psychosomatic complaints as well as trust between the two time points, as seen by the minimum and maximum values of the change measures. The finding that psychosomatic complaints increase with age align with findings from some cross-sectional studies based on the same age groups which reported higher levels of complaints among 17–18 years-olds than among 15–16 years-olds [53, 54] (however, there are also studies which reported fewer complaints in the older age group, e.g., [55]).

The main merit of the current study is the large-scale, longitudinal data material with survey information from a Swedish national sample of adolescents and linked register data on parental characteristics. The use of the FD method for analysing panel data is also a strength, as regressing changes in the dependent variable on changes in the explanatory variables is a fruitful strategy to account for omitted variable bias [49]. Furthermore, the complementary LDV analyses provided empirical support for the assumption that trust is a predictor of psychosomatic complaints, although the reversed analyses showed associations in the opposite direction as well. Nonetheless, there are also limitations. Even though the measures of generalised and institutional trust were based on items recommended by the OECD [47], the scales have not been formally validated (although they were tested and used in an earlier study [48]). It should also be acknowledged that our measure of psychosomatic complaints is based on only three items with limited internal consistency. The measure has a strong somatic component, with two items capturing somatic complaints (headache and stomach ache) and one item reflecting psychological complaints (difficulties falling asleep). Future studies should examine the associations between trust and scales of somatic and psychological complaints, respectively. Additionally, the change in the form of administration of the survey between t1 and t2 could potentially have affected the results. For instance, completion of questionnaires in classrooms may have implied a higher risk of social desirability bias. However, the cross-sectional analyses of t1 and t2 presented very similar results. Furthermore, the non-response in several steps may have compromised the representativeness of the data. For the initial baseline study, about two-thirds of the invited schools agreed to participate [42]. There was also some bias in the attrition at the individual level between the two data collections, implying that the generalisability of the results may be somewhat restricted. Finally, not least since Sweden is a country characterised by comparatively high levels of both generalised and institutional trust [56], it should be acknowledged that the generalisability of the findings to other contexts may be limited. To corroborate the results, studies of generalised and institutional trust and psychosomatic complaints among adolescents also in other national settings are wanted.

Given the clear links between trust and psychosomatic complaints in adolescents, and the importance of trust for a well-functioning and socially sustainable society, a relevant question is how trust can be promoted in young people. Prior studies have identified the school as one important arena, where a school climate characterised by openness, fairness, compassion, and lack of conflicts can enhance trust [23]. Furthermore, experiences of bullying victimisation at school have been shown to be linked with decreases in social trust among students [24]. Hence, a favourable social climate seems beneficial not only for students’ thriving and academic achievement, but also for inducing trust.

In conclusion, this study showed inverse cross-sectional associations between generalised and institutional trust and psychosomatic complaints among adolescents at two time points (ages 15–16 and 17–18 years). Longitudinal analyses applying the FD approach showed that increases in generalised and in institutional trust between ages 15–16 and 17–18 years were associated with corresponding decreases in psychosomatic complaints. The LDV approach provided support for the assumption that higher levels of trust may lead to lower levels of psychosomatic complaints. The reversed LDV analyses additionally showed that psychosomatic complaints were prospectively associated with lower levels of trust. Taken together, the findings suggest that there are reciprocal links between trust and psychosomatic complaints in adolescents. Trust as well as good health and wellbeing are, in turn, central aspects of a socially sustainable society.
