# Supplementary material for: Trust and Psychosomatic Complaints in Adolescence: Findings From a Swedish Cohort Study
Source: Int J Public Health. 2023 Oct 11;68:1606032. doi: 10.3389/ijph.2023.1606032 (PMC10598280; doi:10.3389/ijph.2023.1606032)
Supplement: Supplementary file 1 [file DataSheet1.pdf]

Supplementary Material.

TABLE S1. Descriptives of full t1 sample. n=5537. Futura01 survey, Sweden, 2017 and 2019.

	n	%				
Gender						
Boys	2743	49.5				
Girls	2794	50.5				
Missing	*0*	-				
Family type (t1)						
Two parents	3734	67.4				
One parent	823	14.9				
Shared residence	784	14.2				
Other/Missing	196	3.5				
Missing	*0*	-				
Parental education						
≤2 years secondary or less	993	18.4				
≥3 years secondary	1159	21.4				
Tertiary	3255	60.2				
Missing	*130*	-				
Parental country of birth						
At least one in Sweden	4400	82.1				
At least one in Europe	291	5.4				
Two parents outside Europe	667	12.5				
Missing	*179*	-				
Upper secondary programme (t2)						
Vocational	862	20.8				
Academic	2973	71.8				
Other programme/other activity/missing	307	7.4				
Missing	*1395*	-				
	Mean	s.d.	Min	Max	Skewness	Kurtosis
Psychosomatic complaints						
t1 (n=5450)	7.07	2.78	3	15	0.52	2.54
t2 (n=4076)	7.25	2.73	3	15	0.43	2.48
Change (t2-t1) (n=4025)	0.20	2.47	-10	12	0.06	3.93
Generalised trust						
t1 (n=5367)	2.40	0.51	1	4	-0.18	2.95
t2 (n=3973)	2.42	0.51	1	4	-0.18	2.93
Change (t2-t1) (n=3889)	0.00	0.52	-2.25	1.75	-0.06	3.65
Institutional trust						
t1 (n=5303)	2.78	0.59	1	4	-0.55	3.57
t2 (n=3971)	2.79	0.54	1	4	-0.47	3.52
Change (t2-t1) (n=3852)	-0.02	0.56	-3.0	2.8	0.02	4.68

TABLE S2. Mean values and p-values from ANOVAs of generalised and institutional trust by covariates. n=3691. Futura01 survey, Sweden, 2017 and 2019.

	Generalised trust		Institutional trust	
	t1	t2	t1	t2
	Mean	Mean	Mean	Mean
Gender				
Boys	2.46	2.45	2.80	2.77
Girls	2.37	2.40	2.84	2.82
	p<0.001	*p=0.002*	*p=0.040*	*p=0.003*
Family type (t1)				
Two parents	2.45	2.47	2.85	2.84
One parent	2.28	2.25	2.69	2.65
Shared residence	2.39	2.38	2.80	2.77
Other/Missing	2.29	2.24	2.65	2.67
	p<0.001	*p<0.001*	*p<0.001*	*p<0.001*
Parental education				
≤2 years secondary or less	2.34	2.29	2.65	2.62
≥3 years secondary	2.37	2.35	2.71	2.69
Tertiary	2.44	2.47	2,89	2.87
	p<0.001	*p<0.001*	*p<0.001*	*p<0.001*
Parental country of birth				
At least one in Sweden	2.44	2.46	2.84	2.83
At least one in Europe	2.24	2.24	2.69	2.65
Two parents outside Europe	2.29	2.17	2.73	2.64
	p<0.001	*p<0.001*	*p<0.001*	*p<0.001*
Upper secondary programme (t2)				
Vocational	2.36	2.34	2.59	2.59
Academic	2.43	2.45	2.89	2.87
Other programme/other activity/missing	2.37	2.32	2.56	2.51
	p=0.002	*p<0.001*	*p<0.001*	*p<0.001*
